# Disease-dependent variations in the timing and causes of readmissions in Germany: A claims data analysis for six different conditions

**DOI:** 10.1371/journal.pone.0250298

**Published:** 2021-04-26

**Authors:** Carmen Ruff, Alexander Gerharz, Andreas Groll, Felicitas Stoll, Lucas Wirbka, Walter E. Haefeli, Andreas D. Meid

**Affiliations:** 1 Department of Clinical Pharmacology and Pharmacoepidemiology, Heidelberg University Hospital, Heidelberg, Germany; 2 Faculty of Statistics, TU Dortmund University, Dortmund, Germany; Brown University, UNITED STATES

## Abstract

**Background:**

Hospital readmissions place a major burden on patients and health care systems worldwide, but little is known about patterns and timing of readmissions in Germany.

**Methods:**

We used German health insurance claims (AOK, 2011–2016) of patients ≥ 65 years hospitalized for acute myocardial infarction (AMI), heart failure (HF), a composite of stroke, transient ischemic attack, or atrial fibrillation (S/AF), chronic obstructive pulmonary disease (COPD), type 2 diabetes mellitus, or osteoporosis to identify hospital readmissions within 30 or 90 days. Readmissions were classified into all-cause, specific, and non-specific and their characteristics were analyzed.

**Results:**

Within 30 and 90 days, about 14–22% and 27–41% index admissions were readmitted for any reason, respectively. HF and S/AF contributed most index cases, and HF and COPD accounted for most all-cause readmissions. Distributions and ratios of specific to non-specific readmissions were disease-specific with highest specific readmissions rates among COPD and AMI.

**Conclusion:**

German claims are well-suited to investigate readmission causes if longer periods than 30 days are evaluated. Conditions closely related with the primary disease are the most frequent readmission causes, but multiple comorbidities among readmitted cases suggest that a multidisciplinary care approach should be implemented vigorously addressing comorbidities already during the index hospitalization.

## Introduction

Hospital admissions place a major burden on healthcare systems worldwide and also on Germany with almost 20 million hospitalization cases each year [1, 2]. Absolute hospital admissions are the sum of first admissions and readmissions occurring within a certain interval after the preceding (index) admission. Both, index admission and readmission can be planned admissions or emergency/unforeseen admissions. The latter can be caused by patient factors (e.g. natural course of disease, accidents, non-adherence [3]) and by inadequate care (e.g. underuse [4–6], lacking monitoring of adverse events [5, 7], poorly organized or too early discharge [8–10]). Using raw admission rates for healthcare benchmarking is highly controversial [11–13] and the current practice of considering fixed timeframes of 30 d is rarely scrutinized.

The largest proportion of research on hospital readmissions is provided by data from the United States of America where the Hospital Readmission Reduction Program was introduced in 2012 [14] and hospital readmissions have been considered as events to avert. Whereas in many other countries the knowledge on readmissions for different conditions and populations is continuously increasing (e.g. [15–21]), comparatively little efforts were dedicated to research on readmissions in Germany, the largest European healthcare system, and information on the usability of claims data is particularly limited. Nonetheless, there are conditions of high economical and clinical importance for Germany, generating a high proportion of hospital admissions: The diagnosis of heart failure, for example, was the most frequent discharge diagnosis in German hospitals in 2016, 2017, and 2018 [22]. Atrial fibrillation and atrial flutter was the second most common discharge diagnosis in 2018, but angina pectoris (International Statistical Classification of Diseases and Related Health Problems 10^th^ Revision (ICD-10) code I20) and chronic obstructive pulmonary disease (ICD-10 J44) were also among the ten most common discharge diagnoses. Approximately one quarter of 70-79-year-old women in Germany are diagnosed with osteoporosis [23], so that this disease is also considered common. Especially with regard to the prevention of frailty and the preservation of functional autonomy, the avoidance of (fall-associated) fractures is of utmost importance, to which adequate pharmacotherapy can contribute significantly [24]. The fourth most frequent secondary diagnosis in 2016, 2017, and 2018 of patients treated as inpatients in hospitals was type 2 diabetes mellitus [25], which is diagnosed in about 7% of 18-79-year-olds in Germany [26]; this shows that this clinical picture should also be specifically addressed. All these conditions have in common that they are generally well treatable with drugs and that guidelines with the highest level of evidence exist. The absence of such therapies or problems caused by drugs (e.g. hypoglycemia, kidney failure, electrolyte imbalance, bleeding) can lead to hospital readmission.

In this stage one analysis, we aimed to demonstrate that readmissions for six chronic diseases of high clinical and economic relevance can be validly studied within German health insurance claims and therefore compared two medical conditions with corresponding data reported from the US health care system [27]. To identify patterns and timing of readmissions after an index event in the German population, we analyzed hospital readmissions for six relevant disease entities in a cohort of older people from a large German statutory health insurance, i.e., acute myocardial infarction (AMI), heart failure (HF), a composite of stroke, transient ischemic attack, or atrial fibrillation (S/AF), chronic obstructive pulmonary disease (COPD), type 2 diabetes mellitus (DM), and osteoporosis (OS). With the aim to gain further insight in causes and patterns of readmissions, we explored patterns of all-cause, disease-specific, and non-specific readmissions for each condition and compared frequencies and temporal trends in these patterns.

## Materials and methods

We used health insurance claims (years 2011–2016) from a large German statutory health insurance company (AOK Baden-Württemberg) with overall 4 827 204 beneficiaries and compiled a data base for beneficiaries ≥ 65 years with a complete claims history for the period 2011–2016. While 2011 served as a run-in period, we focused on valid, plausible hospitalization cases as indicated by reimbursement via the diagnosis-related-groups system for the years 2012–2016 (S1 Fig).

The population of beneficiaries ≥ 65 years old was selected for three reasons: (1) The external reference population also referred to the patient population ≥ 65 years old [27]; (2) already 44% of hospital admissions in Germany in 2018 were caused by the group ≥ 65 years old [28], and (3) per capita spending on medical care is significantly higher in this patient group than in the age groups < 65 years [29]. Consequently, a reduction of hospital readmissions would lead to extensive savings of economic resources. On the other hand, these patients are a very vulnerable group, susceptible to adverse drug reactions and a more severe course of disease, especially if they are frail. Identifying the underlying causes for hospital readmissions could help to protect this special group from these detrimental events.

From the clinical perspective, we defined an index admission as a hospital admission that was unequivocally caused by one of the six conditions of interest (AMI, HF, S/AF, COPD, DM, or OS). A readmission case was defined as a second hospital admission that happened within a pre-specified timeframe (i.e., 30 or 90 d) after the index case, yielding index and readmission pairs. All-cause readmission was defined as any readmission case within the specified timeframe, whereas a specific readmission case was directly related to the index case and disease, i.e., a typical complication, exacerbation, or sequel of the index case or its treatment. Non-specific readmission cases were defined as the difference of all-cause and specific readmissions.

In a first step of data preparation, we classified hospital cases independently of diagnoses into eligible index and readmission cases. An index case was required to come from one single hospital with comprehensive information on the patient’s health status (i.e., diagnoses, procedures). The details of readmission cases are specified in the S1 Appendix, which also provide detailed information on the characterization of study outcomes (i.e., hospitalizations) and their comparison with external reference data [27], the procedures to investigate the sequence of index conditions and readmission causes, definitions of reasons for specific readmissions, procedures to assess the most frequent discharge diagnoses, reasons for non-specific 90-d readmissions, and procedures to analyze manifestations and complications of the diabetic foot syndrome. We excluded patients/cases with in-hospital death only if the in-hospital death occurred during the first index admission, therefore, death could be a competing event to hospital readmission.

In Germany, claims data analyses do not require ethics committee approval by law. All data were fully anonymized for the analysts.

### Statistical analysis

Descriptive statistics were used to calculate proportions and ratios of all-cause, specific, and non-specific readmissions, partly aggregated on a weekly basis. Chapters of the International Statistical Classification of Diseases and Related Health Problems 10th Revision (ICD-10) indicating non-specific readmissions were considered relevant if they accounted for ≥ 5% of all readmissions at least once during the observation period.

Differences in readmission frequency over time between the external reference and our data were analyzed by comparing deciles of readmission cases to examine respective times to readmission. To extract necessary information from the external reference [27], we digitized the values behind published graphs using WebPlotDigitizer [30] to obtain the number of readmissions for each post-discharge day.

Data preparation steps were performed using the Microsoft Structured Query Language (MS SQL) Server 2017. Statistical analyses were conducted using the R software environment in version 3.6.0 (R Foundation for Statistical Computing, Vienna, Austria).

## Results

For the time period of 2012–2016, we identified 1 841 877 distinct beneficiaries with documented 5 039 570 hospital cases. After data cleansing and preparation (S1 Fig), 1 689 019 cases remained eligible for analyses, including 569 912 distinct beneficiaries older than 65 years with a complete claims history.

About 14–22% of index admissions were readmitted within 30 d, and 27–41% within 90 d (all-cause readmissions) (Table 1). Varying with the index condition, the same or a related reason for readmission (specific readmissions) was found in 3–13% of index admissions within 30 d, and 5–25% within 90 d. Most index cases were attributed to HF (4.96%) and S/AF (4.99%), and HF and COPD accounted for most of the 30-d (21.6% and 21.0%, respectively) and 90-d (41.0% and 41.2%, respectively) all-cause readmissions. S2 Table shows the most frequent particular diagnoses leading to readmission within 30 d and 90 d; the most frequent discharge diagnosis overall was HF. S3 Table shows the number of specific readmissions for DM with a manifestation or complication of the diabetic foot syndrome, i.e. infection and/or ulceration, peripheral vascular disease, peripheral neuropathy, deformation, or prior amputation(s).

**Table 1:** Number and proportion of hospital admissions and readmissions for the years 2012–2016.

Disease	Nature of admission	Number of cases (%*) with readmission within	
		30 d (absolute and relative)	90 d (absolute and relative)
**COPD**	Index	29323 (100%)	28446 (100%)
	No readmission	23165 (79.0%)	16715 (58.8%)
	All-cause readmission	6158 (21.0%)	11731 (41.2%)
	Specific readmission	3794 (12.9%)	7217 (25.4%)
	Non-specific readmission	2364 (8.1%)	4514 (15.9%)
**Osteoporosis**	Index	6315 (100%)	6111 (100%)
	No readmission	5100 (80.8%)	3951 (64.7%)
	All-cause readmission	1215 (19.2%)	2160 (35.3%)
	Specific readmission	352 (5.6%)	664 (10.9%)
	Non-specific readmission	863 (13.7%)	1496 (24.5%)
**Type 2 diabetes mellitus**	Index	24338 (100%)	23665 (100%)
	No readmission	20164 (82.8%)	15623 (66.0%)
	All-cause readmission	4174 (17.2%)	8042 (34.0%)
	Specific readmission	1290 (5.3%)	2621 (11.1%)
	Non-specific readmission	2884 (11.8%)	5421 (22.9%)
**Heart failure**	Index	83814 (100%)	80870 (100%)
	No readmission	65689 (78.3%)	47678 (59.0%)
	All-cause readmission	18125 (21.6%)	33192 (41.0%)
	Specific readmission	9087 (10.8%)	16749 (20.7%)
	Non-specific readmission	9038 (10.8%)	16443 (20.3%)
**Acute myocardial infarction**	Index	19519 (100%)	18893 (100%)
	No readmission	15764 (80.8%)	11620 (61.5%)
	All-cause readmission	3755 (19.2%)	7273 (38.5%)
	Specific readmission	2042 (10.5%)	4116 (21.8%)
	Non-specific readmission	1713 (8.8%)	3157 (16.7%)
**Stroke, TIA, and atrial fibrillation**	Index	84326 (100%)	81632 (100%)
	No readmission	72952 (86.5%)	59294 (72.6%)
	All-cause readmission	11374 (13.5%)	22338 (27.4%)
	Specific readmission	2458 (2.9%)	4397 (5.4%)
	Non-specific readmission	8916 (10.6%)	17941 (22.0%)
**Total**	Index	247635 (100%)	239617 (100%)
	No readmission	202834 (81.9%)	154881 (64.6%)
	All-cause readmission	44801 (18.1%)	84736 (35.4%)
	Specific readmission	19023 (7.7%)	35764 (14.9%)
	Non-specific readmission	25778 (10.4%)	48972 (20.4%)

*Total may deviate from 100% due to rounding.

### Comparison with external reference

**Fig 1:**
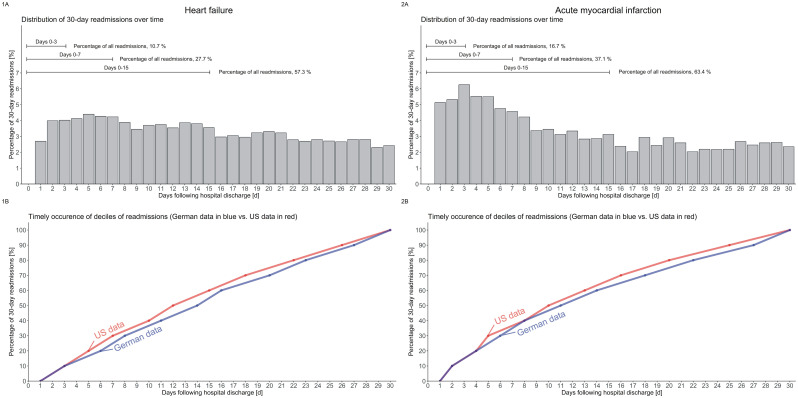
Distribution of 30-d all-cause readmissions after admission for heart failure and acute myocardial infarction, and comparison of their time course with the results of an external reference. Upper panels (1A and 2A) show the distribution of all 30-d readmissions in an older German population for the years 2012–2016. Lower panels (1B and 2B) show the corresponding day of readmission for each decile of hospital readmissions. As examples, for acute myocardial infarction (panel 2B) 30% of readmissions, and for heart failure (panel 1B) 20% of readmissions were reached one day later in the German compared to the US population (day 6 vs. day 5).

In the first 30 d after an index hospitalization for HF or AMI, the distribution of all-cause readmissions of our population closely resembled the patterns reported in Medicare beneficiaries being readmitted (Fig 1). 30-d readmission deciles revealed only marginal differences (lower panels: 1B and 2B, respectively) with slightly later readmission times in the respective deciles of the German population.

### Sequence of index conditions and readmission causes

**Fig 2:**
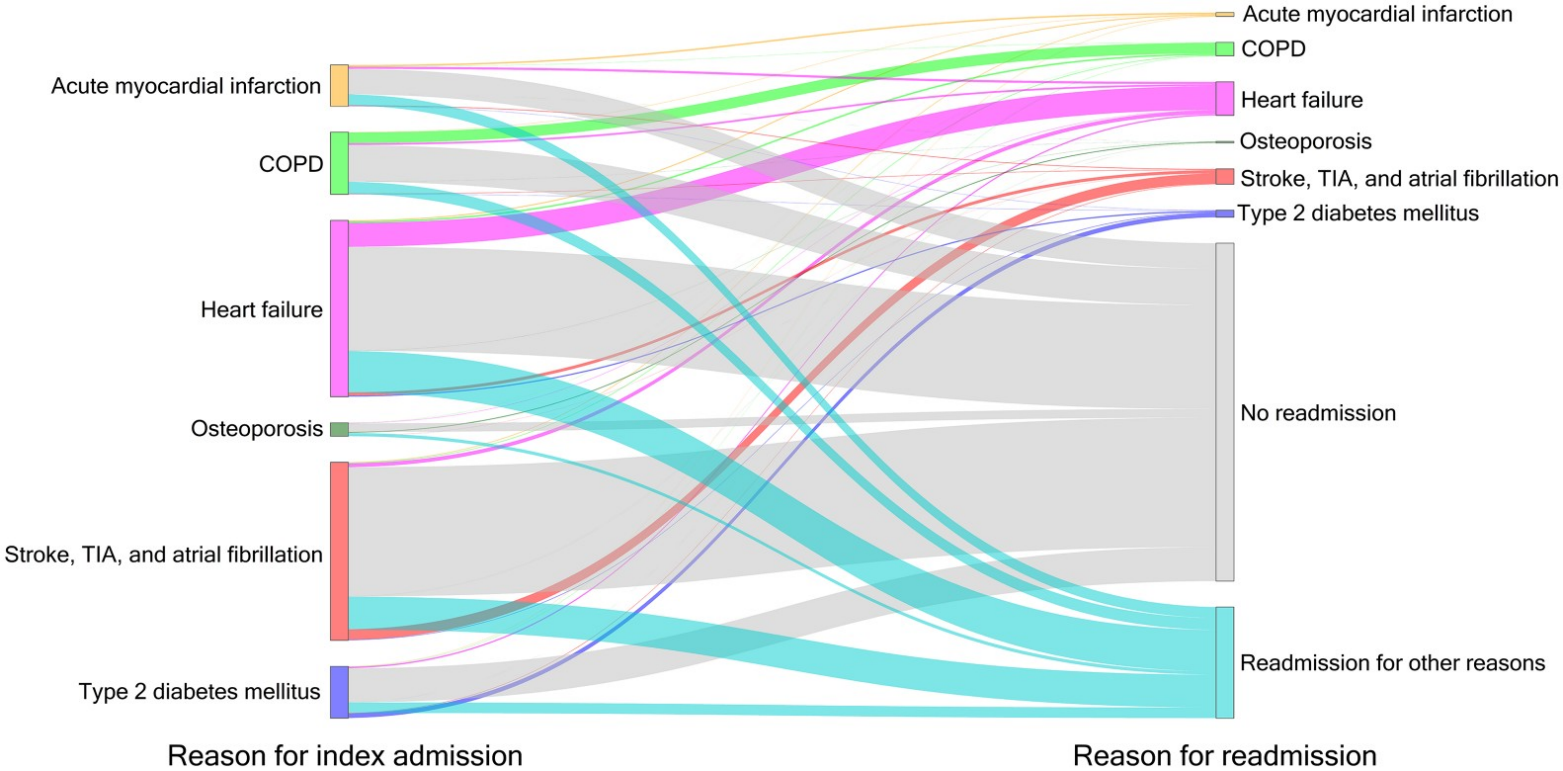
Sankey diagram of index hospitalization and subsequent readmission within 90 d. Colors were assigned to reasons for readmission and the width of the links between nodes (connection between reason for index admission and reason for readmission) and the heights of the nodes (different reasons for admission) indicate the number of corresponding index admission and readmission cases. Looking at cases with an index admission for “heart failure”, the largest proportion of cases was not readmitted (grey link), followed by readmission for other reasons (turquoise link), for heart failure (pink link). The smallest proportion of cases was readmitted for osteoporosis (dark green link).

While most patients were not readmitted within 90 d (64.6%), the remaining fraction was most frequently readmitted for “other reasons for readmission”. The second most frequent reason for readmission was identical with the index condition, except for AMI where the second most frequent reason for readmission was HF (Fig 2). Generally, readmissions for HF represented the most prominent cause of readmission for the analyzed reasons.

### Trajectories of 90-d all-cause or specific readmissions

**Fig 3:**
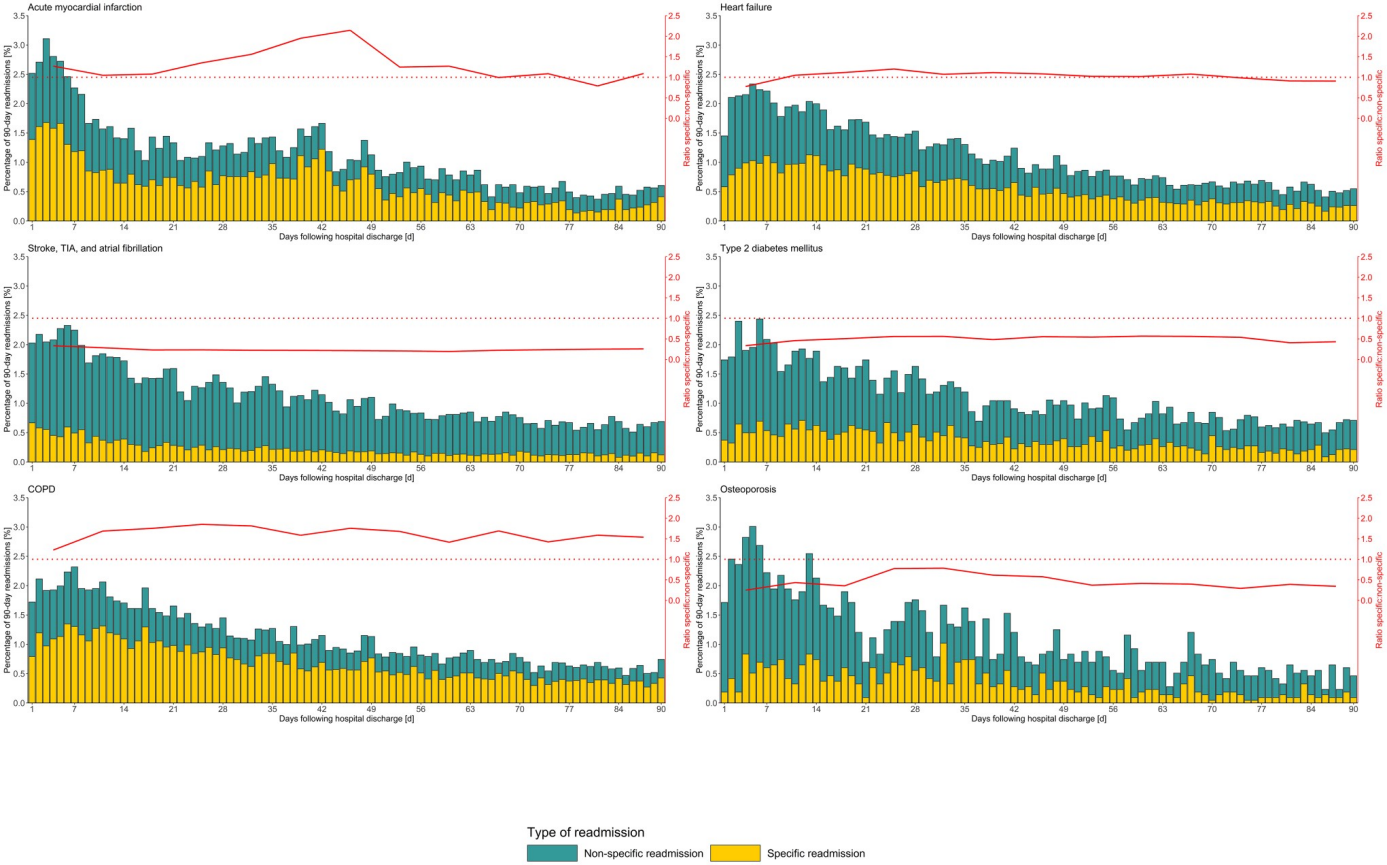
Distributions of all-cause, specific, and non-specific readmissions for six different common diseases. The frequencies of all-cause readmissions are shown by the green bars’ height. Frequencies of specific readmissions are shown in yellow. Ratios between specific and non-specific readmissions are indicated as red solid lines referring to the y-axis on the right side, which shows aggregated weekly values. Ratios > 1 (above dotted red line) indicate a higher number of specific than non-specific readmissions.

The distributions of all-cause and specific readmissions were disease-specific and patterns of trajectories and observed maxima clearly differed (Fig 3). For example, AMI showed a second maximum of readmissions after 30 d. In all six conditions except OS, the curve shape of specific readmissions resembled the curve progression of all-cause readmissions. Disease-specific patterns were also observed in the ratio of specific to non-specific readmissions, which increased between week 3 and week 7 (ratio changes from 1.08 to 2.14) and steeply decreased to 1.25 in week 8 in AMI patients, for example. The patterns of reasons for non-specific readmissions are shown in S2 Fig.

Considering AMI readmissions, about 65% of the specific readmissions between day 21 and day 49 were triggered by ischemic heart disease (ICD-10 I20-I25), 19% by HF (ICD-10 I50, I11.0, I13.0, I13.2, I25.5, I27.9, I42.0), about 3% by bleedings that might be caused by antithrombotic agents (ICD-10 K92.2, K25.0, K29.0, R04.0, K92.1, K25.4, K26.0, K62.5). The most frequent discharge diagnosis within this time period was the diagnosis of coronary 3-vessel disease (ICD-10 I25.13, about 25%) followed by acute subendocardial myocardial infarction (ICD-10 I21.4, about 10%). When identifying procedures that were performed during the readmission for the diagnosis of 3-vessel disease, 89% of cases received either an aortocoronary bypass and/or a coronary angiography (surgeries and medical procedures codes; OPS codes 5–361, 5–362, 5–363.1, 5–363.2, and 8–837).

## Discussion

The findings of our study revealed that approximately 35% of the older German patients are readmitted within 90 d and 18% within 30 d after an index admission. As expected, the causes of readmission depend on the underlying disease [27, 31, 32] and concurrent conditions [33–36], and it can be reasonably assumed that many of them can be prevented [37–39]. As a pioneering step towards prevention strategies, we comprehensively assessed the burden of readmission by highlighting temporal trends of specific and non-specific readmission causes in six clinically and economically relevant conditions, which yielded similar patterns when compared with readmission rates in the United States of America [27].

Previous studies reported similar 30-d readmission rates for HF [31, 32] and AMI [40, 41]. A recent meta-analysis reported a pooled readmission rate for AMI of only 12% [42], but our patients were remarkably older; only one of the 14 included studies exclusively considered a population ≥ 65 and found a readmission rate of 22% [43], which is much closer to our estimate (19.2%). Our results are also consistent with reported 30-d readmission rates for COPD (16.5-22.6%) [31, 32, 44], or DM (14.2-25%) [45–48]. Studies on osteoporotic fractures are heterogeneous regarding population age, examined diagnoses, and timeframe of readmissions [49–53] and they focus rather on surgical options [50], complications, or reoccurrence of hip fractures [49, 51, 53]. In our analysis, we chose a more holistic view and included cases diagnosed with OS (ICD-10 codes M80 and M81) independent of the occurrence of a fracture. Therefore, the reported 30-d readmission rates of 10.6-18.9% [50–52] might not be directly comparable to our 30-d readmission rate (19.2%).

Proportions and ratios for specific vs. non-specific readmissions highly depended on the applied outcome definition (ICD-10 codes). For example, S/AF readmissions excluded the readmission for AF so that patients being readmitted for a recurrent episode of AF or cardioversion were classified as non-specific. This could explain the low observed ratio of specific to non-specific readmissions and the large proportion of remaining non-specific readmissions attributable to diseases of the cardiovascular system (S2 Fig).

We considered readmissions including surgical and medical conditions, chronic conditions and acute events (e.g., AMI), conditions related to the natural course of disease and treatment-related complications and so all aspects together give a manifold picture of readmissions. Therefore it was to be expected that trajectories and underlying causes for readmission might be very heterogeneous. An example for this assumption is the strikingly different time trends of readmission (red line in Fig 3) between conditions. For most diseases, the ratio of non-specific to specific readmission was rather constant throughout the 90-d observation period with some diseases being more likely of being readmitted for other conditions (S/AF, DM, OS), some for the same reason (COPD), and some being equally likely (HF). Interestingly, in AMI patients this relationship was not stable, with specific readmissions peaking 3–7 weeks after the index discharge (accumulating to 21.8% within 90 d). This is remarkable because the usual cut-off for readmission analysis at 30 d does not include this late-occurring peak. Considering that a post-infarction rehabilitation therapy in Germany is limited to a maximum of three weeks for inpatients [54], this peak could indicate that elective surgeries or procedures directly performed after rehabilitation therapy. However, the second most frequent specific discharge diagnosis in this period was recurrent AMI, which needs further exploration. Reasons for recurrent AMI identified in recent studies include a high burden of risk factors and comorbid conditions, highlighting the importance of their best possible pharmacological management [55, 56].

COPD is often accompanied by cardiovascular, metabolic, and musculoskeletal comorbidities [57]. However, any exacerbation of COPD is a disabling and often rapidly progressing problem, which frequently leads to hospital (re)admission. Taking a closer look at individual ICD-10 codes, coproliths or coprostasis (ICD-10 K56.4) represented a frequent readmission cause in COPD, as deemed plausible in COPD patients [58–60]. This observation stresses the importance of detailed assessments of these events because it suggests that timely actions (e.g., promoting physical activity in eligible patients and/or prescribing laxatives) might possibly prevent these readmissions.

According to our data, DM only rarely leads to DM-specific admissions with many other related to cardiovascular diseases, such as HF (S2 Table, S2 Fig). Nevertheless, about 47% of specific readmissions are caused by the diabetic foot syndrome (discharge code E11.74 or E11.75), manifested especially as peripheral vascular disease (S3 Table). Our finding stresses the importance of cardiovascular disease for the clinical course of diabetic patients, as well as the costly complication of diabetic foot syndrome and its preventability, which has been known for a decade [61]. For OS, the picture is less clear. With the manifold causes for OS readmissions, it certainly remains an important task to prevent fractures, e.g., by patient education of risk for falls [62], for example.

The chance to be specifically readmitted (for HF) after an index admission for HF is approximately 50% and thus substantially more frequent than with most other conditions (except COPD and AMI). Non-specific reasons were rather diverse (S2 Fig), summing up to the other 50%. This stresses the burden of comorbidity in HF because of which a multidisciplinary care approach and case-management interventions have been suggested [63].

Among the remaining reasons for non-specific readmissions, the relevant ICD-10 chapters and most frequent discharge diagnoses reflect the most widespread diseases and disease categories in the German population [64, 65]. Fig 2 also confirmed previous findings indicating that reasons for readmissions are manifold [27, 36, 44, 47, 66, 67], that comorbidities play a crucial role as a reason for all-cause readmissions [36, 67], and, thus, that absolute (all-cause) readmission rates are likely not a good indicator of the quality of provided healthcare [11–13].

### Future steps and implications

Based on the introduced methods and database a variety of future analyses is conceivable, among them as a logical first step a comprehensive characterization of the respective patient population. Possible variables that need to be analyzed are e.g. sociodemographic properties, including age and sex, comorbidities, or the use of the health care system, i.e. the number of previous hospitalizations. An analysis of potentially inappropriate medication, medication adherence, or polypharmacy will further complement the description of the population. All these variables have also shown to be predictive for hospital readmissions (e.g. [3, 40, 66, 68, 69] that consequently, the development of a prediction model including these variables is the evident following task. As this model would additionally help to identify risk factors causing readmissions, even more tailored strategies for prevention of these events could be designed by clinicians or policy makers.

### Limitations

Our database consisted of claims data from one regional German health insurance, possibly limiting the generalizability of our results to other populations. However, this insurance company insured most people in this region [70] and its data can be considered as the most representative claims data available. Consistent results with an independent external reference and numerous previous literature reports further support this notion. On the other hand, the results might be specific for Germany, providing equally chances for every citizen to encounter a hospital or use the health care system at all. Limited access to hospital treatments due to an insufficient financial situation of the patient, e.g., does not exist in the analyzed data because statutory health insurance in Germany is required by law to pay for treatments of its beneficiaries, and hospitals bill health insurance for the costs directly without charging the patient first. Nevertheless, it might be imaginable that such a scenario could lead to a higher number of visits of primary health care providers due to the usually lower costs caused by an outpatient treatment or even to a higher mortality rate, and consequently to a lower readmission rate. Generally, regional characteristics or specific treatment programs provided by insurance companies [71] can limit the transferability to other regions or health care systems. The comparison of variability of hospital readmissions within Germany was not feasible for several reasons: (1) research on readmissions in Germany is scarce, especially on claims data (e.g. [72, 73]). (2) In German claims data, there is no evidence on readmissions for our particular selection of conditions that we analyzed within our manuscript. (3) Another prerequisite for a reliable comparability of results is the same definition of outcomes and time-frames. For COPD and stroke, readmission analyses have been recently performed in German claims data [72, 73], but other outcome definitions were applied within these analyses. Another interesting analysis would be the differentiation of readmission rates and causes between different types of hospitals/providers. Patients hospitalized in a hospital specialized in a medical specialty might have different underlying reasons for readmission than patients hospitalized in a non-specialized hospital. With this knowledge, tailored interventions and support could be provided for each hospital type and improve patient care individually.

Second, data preprocessing excluded hospital cases deemed not suitable for analyses, which nevertheless appeared in the reimbursement system (albeit at very low frequency). Third, our definitions for index and readmission code sets were guided by published evidence and expert opinion; we cannot exclude that (ir)relevant codes might have been missed or included by mistake, although we meticulously aimed to avoid them by considering multiple sources of information.

### Conclusions

Because the large majority of patients are not readmitted within 90 d, we might assume that medical care in Germany is at a high quality level. Knowing that still about one third of patients (if patients are admitted for HF, COPD, or AMI even about 40% of patients) are readmitted within this timeframe, and knowing that about half of the readmissions occurred within 30 d when considering a follow-up of 90 d, we need to perform three action points: 1. To start analyzing whether readmissions are planned and therefore cannot be prevented. 2. To stop focusing solely on raw readmission rates because manifold reasons can trigger readmissions, which are not indicators of quality of hospital care. 3. To implement strategies avoiding preventable, rapidly recurring, drastic events for patients and the health care system. A consequent preparation and education of the patient to his new life situation and medication at discharge is crucial but a “one-size-fits-all” solution does not exist and is not promising; while for COPD and AMI disease-specific measures may especially reduce the burden of readmission for the same or related reasons, a more holistic concept considering all (and foremost cardiovascular) comorbidities might be suitable for HF, OS, DM, and S/AF.

## Supporting information

S1 Fig Flowchart describing the data cleansing process and reasons for exclusion of hospital cases. DRG: diagnosis-related group.
(PDF)

S2 Fig Distributions of non-specific readmissions for six different conditions according to their ICD-10 chapters. Proportions of the ICD-10 chapters with frequencies ≥ 5% are shown on an aggregated weekly basis. Lines with the same color always represent the same ICD chapter. For each condition analyzed, the proportions of the following ICD-10 chapters reached at least once 5% of all readmissions at each analyzed time point: IX (diseases of the circulatory system) as the generally largest category and XI (diseases of the digestive system). Thus, non-specific COPD readmissions included only these two categories, while OS patterns were much more diverse with eight ICD-10 chapters exceeding 5% prevalence, for example.
(TIFF)

S1 Table ICD-10 codes of index and readmission code-sets for individual disease entities. Notes: As a general principle, index codes also accounted for specific readmission codes, except for S/AF, where codes indicating atrial fibrillation were not used to identify a readmission case, but rather its adverse consequences, such as TIA or stroke. The index condition S/AF was defined as a composite of atrial fibrillation, TIA, and stroke and the sequelae or complications of a stroke cannot be a hospitalization for atrial fibrillation.
(DOCX)

S2 Table Number and proportions of the most frequent discharge diagnoses. Notes: Across most conditions, the most frequent reasons for 30-d and 90-d readmissions are similar, independent of their classification as specific, non-specific, or all-cause readmission, except for AMI and HF. For these two conditions, the most frequent discharge diagnoses for all-cause readmissions and specific readmissions within 30 d differ from those within 90 d. The most frequent all-cause readmission discharge diagnoses are always diagnoses that also indicate a specific readmission, except for S/AF, where the ICD-10 codes for AF were not assigned to the specific readmission code set.
(DOCX)

S3 Table Number and proportions of secondary diagnosis belonging to the diabetic foot syndrome. Notes: The table indicates the number of cases and its proportion having coded one or more manifestations of the diabetic foot syndrome. The existence of the manifestation of complication of the diabetic foot syndrome is indicated by a “1” in the respective field, the non-existence by a “0”, respectively. 276 cases had a diagnosis code belonging to the manifestation of peripheral vascular disease and no other diagnosis code belonging to other manifestations of the diabetic foot syndrome, e.g. 264 cases had a diagnosis code belonging to the manifestation of peripheral vascular disease and simultaneously a diagnosis code belonging to the manifestation of peripheral neuropathy. Total may deviate from 100% due to rounding.
(DOCX)

S1 Appendix Supplementary material and methods. (DOCX)
