# Supplementary figures and images for: Disease-dependent variations in the timing and causes of readmissions in Germany: A claims data analysis for six different conditions
Source: PLoS One. 2021 Apr 26;16(4):e0250298. doi: 10.1371/journal.pone.0250298 (PMC8075250; doi:10.1371/journal.pone.0250298)

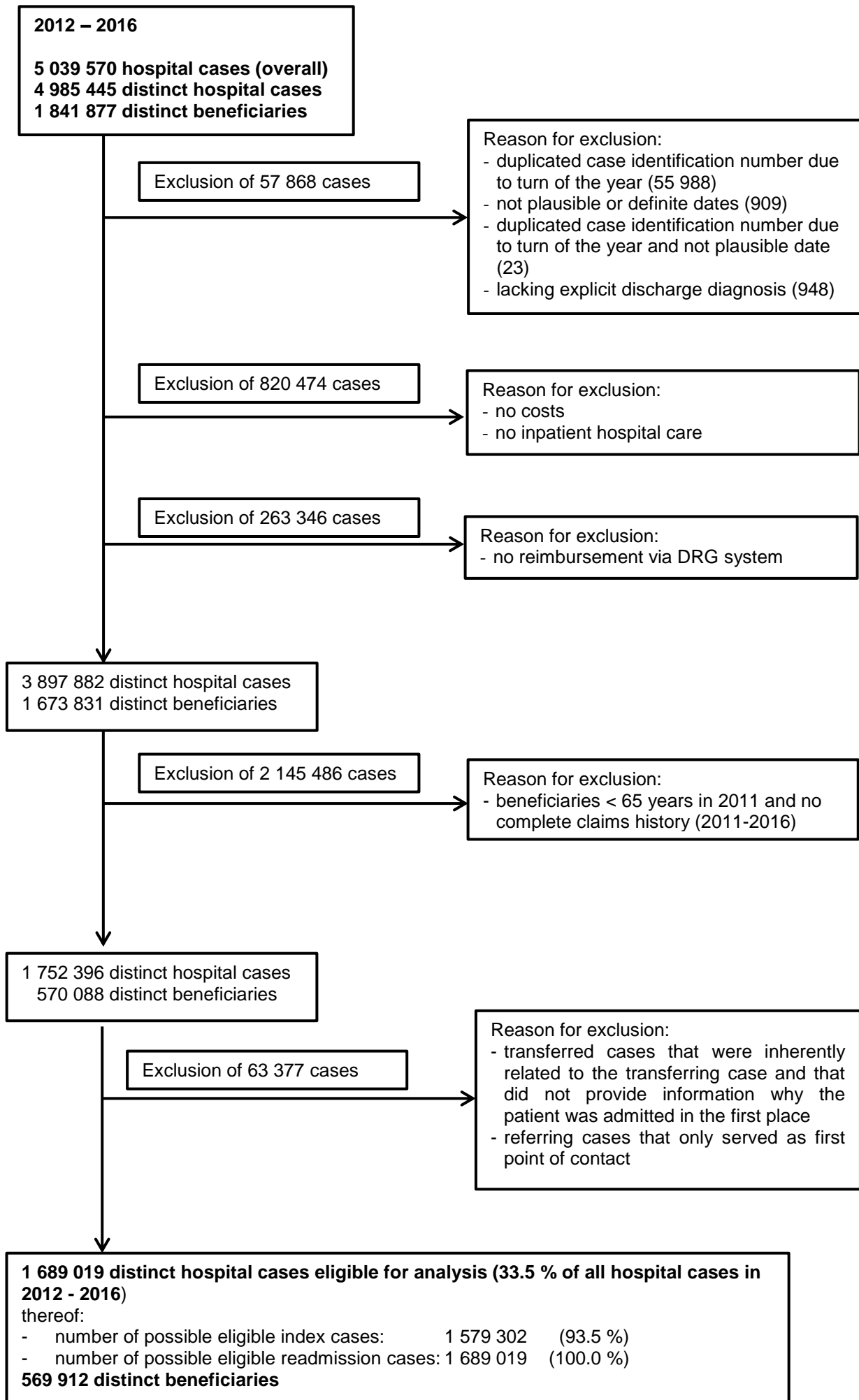

Supplement: S1 Fig — DRG: diagnosis-related group. (PDF) [file pone.0250298.s001.pdf]
