# Supplementary material for: Disease-dependent variations in the timing and causes of readmissions in Germany: A claims data analysis for six different conditions
Source: PLoS One. 2021 Apr 26;16(4):e0250298. doi: 10.1371/journal.pone.0250298 (PMC8075250; doi:10.1371/journal.pone.0250298)
Supplement: S1 Table — Notes: As a general principle, index codes also accounted for specific readmission codes, except for S/AF, where codes indicating atrial fibrillation were not used to identify a readmission case, but rather its adverse consequences, such as TIA or stroke. The index condition S/AF was defined as a composite of atrial fibrillation, TIA, and stroke and the sequelae or complications of a stroke cannot be a hospitalization for atrial fibrillation. (DOCX) [file pone.0250298.s003.docx]

| **Disease** | **Index code-set** | **Readmission code-set** | | | |
| --- | --- | --- | --- | --- | --- |
|  |  | **Index codes that could also indicate a readmission** | **Symptom/**  **manifestation of index disease** | **Sequelae/**  **complication** | **Adverse drug reactions** |
| **COPD** | J44.0-, J44.1-, J44.8-, J44.9- | J44.0-, J44.1-, J44.8-, J44.9- | J96.0-, J96.1-, J96.9-, R06.0, R60.0, R60.1, R60.9, J41.0, J41.1, J41.8, J42 | J96.0-, J96.1-, J96.9-, C34.0, C34.1, C34.2, C34.3, C34.8, C34.9, R06.0, R60.0, R60.1, R60.9, I27.9, I50.01, I50.02, I50.03, I50.04, I50.05, J43.1, J43.2, J43.8, J43.9, J41.0, J41.1, J41.8, J42 | B37.0, R00.0, R68.8 |
| **Osteoporosis** | M80.0-, M80.1-, M80.2-, M80.3-, M80.4-, M80.5-, M80.8-, M80.9-, M81.0-, M81.1-, M81.2-, M81.3-, M81.4-, M81.5-, M81.6-, M81.8-, M81.9- | M80.0-, M80.1-, M80.2-, M80.3-, M80.4-, M80.5-, M80.8-, M80.9-, M81.0-, M81.1-, M81.2-, M81.3-, M81.4-, M81.5-, M81.6-, M81.8-, M81.9- | S22.0-, S22.1, S32.0-, S32.1, S32.2, S32.3, S32.4, S32.5, S32.7,  S32.8-, S42.2-, S42.3, S42.4-, S52.0-, S52.1-, S52.2-, S52.3-, S52.4, S52.5-, S52.6, S52.7, S52.8, S52.9, S72.0-, S72.1- | Z47.0, Z47.8, Z47.9, M84.0-, M84.1-, M84.2- | M87.18, E83.58, E83.59 |
| **Type 2 diabetes mellitus** | E11.0-, E11.1-, E11.2-, E11.3-, E11.4-, E11.5-, E11.6-, E11.7-, E11.8-, E11.9- | E11.0-, E11.1-, E11.2-, E11.3-, E11.4-, E11.5-, E11.6-, E11.7-, E11.8-, E11.9- | E16.0, E16.1, E16.2 | E16.0, E16.1, E16.2 | E87.2, B37.3, B37.4, T38.3, T88.6 |
| **Heart failure** | I50.0-, I50.1-, I50.9, I11.0-, I13.0-, I13.2-, I25.5, I27.9, I42.0 | I50.0-, I50.1-, I50.9, I11.0-, I13.0-, I13.2-, I25.5, I27.9, I42.0 | I95.0, I95.1, I95.2, I95.8, I95.9, I47.1, I47.2, I47.9, R00.0, R06.0, R60.0, R60.1, R60.9, R57.0 | I46.0, I46.1, I46.9, J90, J94.8, R16.0, K76.1, I34.0, I36.1, R57.0, I49.0, I20.-, I21.-, I22.-, Z45.01, Z45.02, Z45.08 | I49.3, I44.0, I44.1, I44.2, R00.1, H53.5, E87.7, E87.1, E87.5, I95.0, I95.1, I95.2, I95.8, I95.9, T46.0, T46.4, T44.7, T50.0, T50.1, T50.2 |
| **Heart failure (comparison with external reference)** | I50.0-, I50.1-, I50.9, I11.0-, I13.0-, I13.2-  (ICD-9-CM codes were translated to ICD-10-GM codes) | No readmission code-set was defined because readmissions for any reason (all-cause readmission) were considered. | | | |
| **Acute myocardial infarction** | I21.0, I21.1, I21.2, I21.3, I21.4, I21.9, I22.0, I22.1, I22.8, I22.9, I23.0, I23.1, I23.2, I23.3, I23.4, I23.5, I23.6, I23.8 | I21.0, I21.1, I21.2, I21.3, I21.4, I21.9, I22.0, I22.1, I22.8, I22.9, I23.0, I23.1, I23.2, I23.3, I23.4, I23.5, I23.6, I23.8 | I20.0, I20.1, I20.8, I20.9, I24.8, I24.9, I25.0, I25.1-, I25.8, I25.9, R57.0 | R00.1, I95.-, I50.0-, I50.1-, I50.9, I49.0, I46.0, I46.1, I24.1, I25.3, I25.5, R57.0 | R00.1, I95.-, G72.0, E87.5, R04.0, R31, K06.8, K92.0, K92.1, K92.2, K29.0, K25.0, K25.1, K25.2, K25.3, K25.4, K25.5, K25.6, K25.7, K25.9, R23.2, T46.1, T46.3, T46.4, T44.7, T50.0 |
| **Acute myocardial infarction (comparison with external reference)** | I21.0, I21.1, I21.2, I21.3, I21.4, I21.9 (ICD-9-CM codes were translated to ICD-10-GM codes) | No readmission code-set was defined because readmissions for any reason (all-cause readmission) were considered. | | | |
| **Stroke, TIA, and atrial fibrillation** | I63.0, I63.1, I63.2, I63.3, I63.4, I63.5, I63.6, I63.8, I63.9, I64, G45.0-, G45.1-, G45.2-, G45.3-, G45.4-, G45.8-, G45.9-, I48.0, I48.1, I48.2, I48.3, I48.4, I48.9 | I63.0, I63.1, I63.2, I63.3, I63.4, I63.5, I63.6, I63.8, I63.9, I64, G45.0-, G45.1-, G45.2-, G45.3-, G45.4-, G45.8-, G45.9- |  | I69.3, I69.4, G81.0, G81.1, G81.9, G82.0-, G82.1-, G82.2-, G82.3-, G82.4-, G82.5-, G83.1, G83.2, G83.3, G83.6, G40.1, G40.2, H53.2, H53.3, H53.4, R27.0, R29.5, R47.0, R47.1, R48.0, R48.1, R48.2, R48.8, R13.0, R13.1, R13.9, J69.0, F01.0, F01.1, F01.2, F01.3, F01.8, F01.9, I65.0, I65.1, I65.2, I65.3, I65.8, I65.9, I66.0, I66.1, I66.2, I66.3, I66.4, I66.8, I66.9 | R04.0, R31, K06.8, K92.0, K92.1, K92.2, K29.0, K25.0, K25.1, K25.2, K25.3, K25.4, K25.5, K25.6, K25.7, K25.9, S06.4, I31.2, R04.1, R04.2, R04.8, R04.9, I85.0, K26.0, K26.2, K26.4, K26.6, K27.0, K27.2, K27.4, K27.6, K28.0, K28.2, K28.4, K28.6, K29.0, K62.5, N93.0, N93.8, N93.9, I60.0, I60.1, I60.2, I60.3, I60.4, I60.5, I60.6, I60.7, I60.8, I60.9, I61.0, I61.1, I61.2, I61.3, I61.4, I61.5, I61.6, I61.8, I61.9, I62.0-, I62.1, I62.9, K22.81, D68.33, D68.34, D68.35, R58, N92.0, N92.1, N92.4, G72.0, E87.1, E87.5, I95.-, T46.4, T50.0, T50.1, T50.2 |

**S1 Table. ICD-10 codes of index and readmission code-sets for individual disease entities**
