# Supplementary material for: Disease-dependent variations in the timing and causes of readmissions in Germany: A claims data analysis for six different conditions
Source: PLoS One. 2021 Apr 26;16(4):e0250298. doi: 10.1371/journal.pone.0250298 (PMC8075250; doi:10.1371/journal.pone.0250298)
Supplement: S2 Table — Notes: Across most conditions, the most frequent reasons for 30-d and 90-d readmissions are similar, independent of their classification as specific, non-specific, or all-cause readmission, except for AMI and HF. For these two conditions, the most frequent discharge diagnoses for all-cause readmissions and specific readmissions within 30 d differ from those within 90 d. The most frequent all-cause readmission discharge diagnoses are always diagnoses that also indicate a specific readmission, except for S/AF, where the ICD-10 codes for AF were not assigned to the specific readmission code set. (DOCX) [file pone.0250298.s004.docx]

|  | **Most frequent discharge diagnoses** | | | | | | | |
| --- | --- | --- | --- | --- | --- | --- | --- | --- |
|  | **of specific readmissions** | |  | **of all-cause readmissions** | |  | **of non-specific readmissions** | |
| **Disease** | **within 30 d (absolute and relative)** | **within 90 d (absolute and relative)** |  | **within 30 d (absolute and relative)** | **within 90 d (absolute and relative)** |  | **within 30 d (absolute and relative)** | **within 90 d (absolute and relative)** |
| **COPD** | **J44.19**  (Chronic obstructive pulmonary disease with acute exacerbation, unspecified: unspecified forced expiratory volume in one second)  (462 readmissions ≡ 12.2 % of all specific readmissions) | **J44.19**  (Chronic obstructive pulmonary disease with acute exacerbation, unspecified: unspecified forced expiratory volume in one second) (844 readmissions ≡ 11.7 % of all specific readmissions) |  | **J44.19**  (Chronic obstructive pulmonary disease with acute exacerbation, unspecified: unspecified forced expiratory volume in one second)  (462 readmissions ≡ 7.5 % of all readmissions) | **J44.19**  (Chronic obstructive pulmonary disease with acute exacerbation, unspecified: unspecified forced expiratory volume in one second) (844 readmissions ≡ 7.2 % of all readmissions) |  | **I50.14**  (Left ventricular failure: with complaints at rest) (101 readmissions ≡ 4.3 % of all non-specific readmissions) | **I50.14** (Left ventricular failure: with complaints at rest) (178 readmissions ≡ 3.9 % of all non-specific readmissions) |
| **Osteoporosis** | **M80.08**  (Postmenopausal osteoporosis with pathological fracture, other sites: head, neck, ribs, skull, trunk, vertebral column) (101 readmissions ≡ 28.7 % of all specific readmissions) | **M80.08** (Postmenopausal osteoporosis with pathological fracture, other sites: head, neck, ribs, skull, trunk, vertebral column)  (192 readmissions ≡ 28.9 % of all specific readmissions) |  | **M80.08**  (Postmenopausal osteoporosis with pathological fracture, other sites: head, neck, ribs, skull, trunk, vertebral column) (101 readmissions ≡ 8.3 % of all readmissions) | **M80.08** (Postmenopausal osteoporosis with pathological fracture, other sites: head, neck, ribs, skull, trunk, vertebral column)  (192 readmissions ≡ 8.9 % of all readmissions) |  | **I50.01** (Secondary right-sided heart failure) (29 readmissions ≡ 3.4 % of all non-specific readmissions) | **I50.01** (Secondary right-sided heart failure) (58 readmissions ≡ 3.9 % of all non-specific readmissions) |
| **Type 2 diabetes mellitus** | **E11.74** (Type 2 diabetes mellitus, with multiple complications, with diabetic foot disease, not decompensated)  (462 readmissions ≡ 35.8 % of all specific readmissions) | **E11.74** (Type 2 diabetes mellitus, with multiple complications, with diabetic foot disease, not decompensated)  (927 readmissions ≡ 35.4 % of all specific readmissions) |  | **E11.74** (Type 2 diabetes mellitus, with multiple complications, with diabetic foot disease, not decompensated)  (462 readmissions ≡ 11.1 % of all readmissions) | **E11.74** (Type 2 diabetes mellitus, with multiple complications, with diabetic foot disease, not decompensated)  (927 readmissions ≡ 11.5 % of all readmissions) |  | **I50.14**  (Left ventricular failure: with complaints at rest) (145 readmissions ≡ 5.0 % of all non-specific readmissions) | **I50.14** (Left ventricular failure: with complaints at rest)  (241 readmissions ≡ 4.4 % of all non-specific readmissions) |
| **Heart failure** | **I50.14** (Left ventricular failure: with complaints at rest)  (2181 readmissions ≡ 24.0 % of all specific readmissions) | **I50.01**  (Secondary right-sided heart failure) (4162 readmissions ≡ 24.8 % of all specific readmissions) |  | **I50.14** (Left ventricular failure: with complaints at rest)  (2181 readmissions ≡ 12.0 % of all readmissions) | **I50.01**  (Secondary right-sided heart failure) (4162 readmissions ≡ 12.5 % of all readmissions) |  | **E86**  (Volume depletion) (266 readmissions ≡ 2.9 % of all non-specific readmissions) | **E86** (Volume depletion) (425 readmissions ≡ 2.6 % of all non-specific readmissions) |
| **Acute myocardial infarction** | **I21.4**  (Acute subendocardial myocardial infarction) (346 readmissions ≡ 16.9 % of all specific readmissions) | **I25.13**  (Atherosclerotic heart disease, 3-vessel-disease) (652 readmissions ≡ 15.8 % of all specific readmissions) |  | **I21.4**  (Acute subendocardial myocardial infarction) (346 readmissions ≡ 9.2 % of all readmissions) | **I25.13**  (Atherosclerotic heart disease, 3-vessel-disease) (652 readmissions ≡ 9.0 % of all readmissions) |  | **R55**  (Syncope and collapse) (41 readmissions ≡ 2.4 % of all non-specific readmissions) | **R55** (Syncope and collapse)  (69 readmissions ≡ 2.2 % of all non-specific readmissions) |
| **Stroke, TIA, and atrial fibrillation** | **I63.4** (Cerebral infarction due to embolism of cerebral arteries) (218 readmissions ≡ 8.9 % of all specific readmissions) | **I63.4** (Cerebral infarction due to embolism of cerebral arteries) (428 readmissions ≡ 9.7 % of all specific readmissions) |  | **I48.0**  (Paroxysmal atrial fibrillation) (666 readmissions ≡ 5.9 % of all readmissions) | **I48.0**  (Paroxysmal atrial fibrillation) (1429 readmissions ≡ 6.4 % of all readmissions) |  | **I48.0** (Paroxysmal atrial fibrillation) (655 readmissions ≡ 7.3 % of all non-specific readmissions) | **I48.0**  (Paroxysmal atrial fibrillation) (1416 readmissions ≡ 7.9 % of all non-specific readmissions) |

**S2 Table. Number and proportions of the most frequent discharge diagnoses.**
