# Supplementary material for: Disease-dependent variations in the timing and causes of readmissions in Germany: A claims data analysis for six different conditions
Source: PLoS One. 2021 Apr 26;16(4):e0250298. doi: 10.1371/journal.pone.0250298 (PMC8075250; doi:10.1371/journal.pone.0250298)
Supplement: S3 Table — Notes: The table indicates the number of cases and its proportion having coded one or more manifestations of the diabetic foot syndrome. The existence of the manifestation of complication of the diabetic foot syndrome is indicated by a “1” in the respective field, the non-existence by a “0”, respectively. 276 cases had a diagnosis code belonging to the manifestation of peripheral vascular disease and no other diagnosis code belonging to other manifestations of the diabetic foot syndrome, e.g. 264 cases had a diagnosis code belonging to the manifestation of peripheral vascular disease and simultaneously a diagnosis code belonging to the manifestation of peripheral neuropathy. Total may deviate from 100% due to rounding. (DOCX) [file pone.0250298.s005.docx]

**S3 Table. Number and proportions of secondary diagnosis belonging to the diabetic foot syndrome.**

| **Infection and/or ulceration** | **Peripheral vascular disease** | **Peripheral neuropathy** | **Deformities** | **Prior amputation(s)** | **Absolute number of cases** | **Proportion of cases with secondary diagnosis for diabetic foot manifestation or complication [%]** |
| --- | --- | --- | --- | --- | --- | --- |
| 0 | 1 | 0 | 0 | 0 | 276 | 24,08 |
| 0 | 1 | 1 | 0 | 0 | 264 | 23,04 |
| 1 | 1 | 0 | 0 | 0 | 130 | 11,34 |
| 1 | 1 | 1 | 0 | 0 | 116 | 10,12 |
| 1 | 0 | 0 | 0 | 0 | 58 | 5,06 |
| 0 | 1 | 1 | 0 | 1 | 58 | 5,06 |
| 0 | 1 | 0 | 0 | 1 | 45 | 3,93 |
| 0 | 0 | 1 | 0 | 0 | 42 | 3,66 |
| 1 | 1 | 1 | 0 | 1 | 39 | 3,40 |
| 1 | 1 | 0 | 0 | 1 | 33 | 2,88 |
| 1 | 0 | 1 | 0 | 0 | 22 | 1,92 |
| 1 | 0 | 0 | 0 | 1 | 11 | 0,96 |
| 0 | 0 | 0 | 0 | 1 | 9 | 0,79 |
| 1 | 0 | 1 | 0 | 1 | 8 | 0,70 |
| 1 | 0 | 1 | 1 | 1 | 8 | 0,70 |
| 0 | 0 | 1 | 0 | 1 | 7 | 0,61 |
| 1 | 1 | 1 | 1 | 1 | 4 | 0,35 |
| 1 | 1 | 1 | 1 | 0 | 3 | 0,26 |
| 0 | 1 | 0 | 1 | 0 | 3 | 0,26 |
| 1 | 0 | 1 | 1 | 0 | 3 | 0,26 |
| 1 | 1 | 0 | 1 | 0 | 2 | 0,17 |
| 1 | 1 | 0 | 1 | 1 | 1 | 0,09 |
| 1 | 0 | 0 | 1 | 0 | 1 | 0,09 |
| 0 | 1 | 1 | 1 | 0 | 1 | 0,09 |
| 0 | 0 | 1 | 1 | 0 | 1 | 0,09 |
| 0 | 0 | 0 | 1 | 1 | 1 | 0,09 |
| **Total** | | | | | **1146** | **100 %** |
