# Supplementary material for: Disease-dependent variations in the timing and causes of readmissions in Germany: A claims data analysis for six different conditions
Source: PLoS One. 2021 Apr 26;16(4):e0250298. doi: 10.1371/journal.pone.0250298 (PMC8075250; doi:10.1371/journal.pone.0250298)
Supplement: S1 Appendix — (DOCX) [file pone.0250298.s006.docx]

**Supplementary Material and Methods**

**Determination of readmission cases beyond index cases**

To ensure complete data collection, cases were only eligible as index cases if they spent the entire inpatient stay in the same hospital. As soon as a patient is transferred from one hospital to another hospital, it must be assumed that the information about the entire duration of the hospital stay cannot be found in only one case but rather in two different cases, namely the transferring case and the transferred case. These cases can therefore not be considered index cases. Each eligible index case was also considered to be an eligible readmission case. A readmission case was additionally defined as a hospital case that transferred the patient to another second hospital (“transferring case” = “Verlegungsfall” or “Fall des verlegenden Krankenhauses”) and therefore provided information for the reason of admission in the first place (admission or discharge diagnosis), but lacked comprehensive information on the entire treatment period of the actual condition. A hospital case was also considered as readmission case if the case received care in one single hospital but lacked comprehensive information due to only short periods of time spent at home (“Fallzusammenführung aufgrund von Wiederaufnahme in dasselbe Krankenhaus”; this type of hospital case is specifically marked in the claims data and can therefore easily be identified).

Transferred cases were not considered for the analysis because they are inherently related to the transferring case and do not provide information on the reason why the patient was admitted in the first place (“transferred case” = “Fall des aufnehmenden Krankenhauses”). Conversely, if the transferring case serves only as first point of contact for the patient and lasts only few hours (“Stundenfall”) because the patient was transferred to another hospital on the same day, then this transferring case was not considered and the transferred case that subsequently followed was considered as readmission case providing admission and discharge diagnosis. Of note, the number of transferring cases that were eligible for analysis is 50 902, leading to a ratio of 3.01 % (i.e. (50 902/1 689 019)*100 %)) of distinct hospital cases eligible for analysis, i.e. a rather small proportion, which was considered unlikely to have affected the results to a relevant extent.

**Outcome (hospitalizations)**

For each of the six conditions of interest (AMI, HF, S/AF, COPD, DM, and OS), we specified one code set of ICD-10 codes representing diagnoses for index cases and another code set representing diagnoses for specific readmission cases. The complete compilation of ICD-10 codes is provided in S1 Table. For each condition, we applied the index code-set to eligible cases and considered the hospitalization as an index case if the discharge diagnosis was identical with a listed ICD-10 index code. Conversely, the hospitalization was considered to represent a specific readmission case if the discharge diagnosis or the admitting diagnosis was identical with one of the specified ICD-10 readmission codes.

**Comparison with external reference data**

As an external reference, we chose the work of Dharmarajan and co-workers [1], translated the given ICD-9 codes to ICD-10 index codes for HF and AMI (consistent with German billing codes), excluded patients/cases with in-hospital death only if the in-hospital death occurred during the first index admission, did not exclude cases with discharge against medical advice, allowed hospitalizations occurring within a 30-d period after the index case to count as new index hospitalization, and defined that a new case could only start on the day after the discharge day of the preceding case.

**Sequence of index conditions and readmission causes**

To illustrate the chronological sequence of analyzed conditions in a Sankey diagram, we classified readmissions following an index admission as readmission for AMI, HF, S/AF, COPD, DM, or OS (according to the corresponding index-code set), as readmission for other reasons, or as ”no readmission” within 90 d. Our readmission code-sets contain ICD-10 codes indicating specific adverse drug reactions or sequelae, which are meaningful in direct connection with the underlying index disease but might be misleading if analyzed independently. As an example, a diagnosis code for bleeding might be highly specific for an adverse drug reaction of anticoagulant or antiplatelet therapy after stroke but stroke is not an obvious reason for readmission due to bleeding after an index admission for COPD. The index code sets are unequivocally related to the condition and therefore not prone to misinterpretation when analyzing the sequence of different conditions.

**Reasons for specific readmissions**

A specific readmission was defined as a hospitalization that is potentially clinically related to the immediately preceding index hospitalization, i.e., it had to be in conjunction with the diagnosis or related symptoms of the index disease, sequelae, or adverse drug reactions due to drug therapy for the index condition.

ICD-10 codes for the index code-set were selected on the basis of published codes (S/AF: [2-4], AMI: [1, 5, 6], HF: [1, 7, 8], DM: [9-11], COPD: [12-14], OS: [15-17]). ICD-10 codes for the specific readmission code-set were chosen according to published literature, guidelines (sequelae or complications), and the summary of product characteristics (adverse drug reactions) provided that they were specific for the respective disease or therapy. Two physicians reviewed the selected code sets and the sets were accordingly adapted.

**Reasons for non-specific 90-d readmissions**

After removing cases with “specific readmission” from all-cause readmissions, the remaining reasons for non-specific readmission were characterized by their discharge diagnosis and related ICD-10 chapters. This analysis was performed to obtain an overview of additional disease groups that play an important role within a defined timeframe for each index condition; if these groups differed between index conditions.

**Most frequent discharge diagnoses**

To analyze the most frequent diagnoses of readmissions for each condition of interest within 30 d and 90 d, we focused on the discharge diagnosis because it is the diagnosis that defines the main condition for which the patient was treated in the hospital. Furthermore, there is exactly one discharge diagnosis per hospital case, which enables us to descriptively analyze proportions on a case-related basis and thus in a transparent way. Frequencies of discharge diagnoses were described as a percentage of all readmissions, analyzing the most frequent reasons of all-cause, specific, and non-specific readmissions.

**Manifestations or complications of the diabetic foot syndrome**

According to the German coding references (“Deutsche Kodierrichtlinien”) that must be applied when coding diagnoses of the ICD-system in Germany, the diabetic foot must be coded by the ICD-codes E11.74 or E11.75 and each manifestation or complication of the diabetic foot must be coded as a secondary diagnosis if applicable [18]. The coding references also make suggestions which codes may belong to the diabetic foot and classify them into five categories: “infection and/or ulceration” (ICD-10-GM codes L02.4, L03.02, L03.11, L89.0-, L89.1-, L89.2-, L89.3-, L89.9-, L97), “peripheral vascular disease” (ICD‑10‑GM codes I70.20, I70.21, I70.22, I70.23, I70.24, I70.25, I70.29), “peripheral neuropathy” (ICD-10-GM codes G63.2*, G99.0*), “deformities” (ICD‑10‑GM codes M20.1, M20.2, M20.3, M20.4, M20.5, M21.27, M21.37, M21.4, M21.57, M21.6-), “prior amputation(s)” (ICD-10-GM codes Z89.4, Z89.5, Z89.6, Z89.7). We analyzed the secondary diagnoses of each readmission case which had a discharge diagnosis of diabetic foot complications (ICD-10-GM codes E11.74 or E11.75) and calculated numbers and proportions of readmission cases according to the five categories of diabetic foot syndrome indicated by the German coding references.

**References**

1. Dharmarajan K, Hsieh AF, Lin Z, Bueno H, Ross JS, Horwitz LI, et al. Diagnoses and timing of 30-day readmissions after hospitalization for heart failure, acute myocardial infarction, or pneumonia. JAMA. 2013;309(4):355-63. doi: 10.1001/jama.2012.216476. PubMed PMID: 23340637; PubMed Central PMCID: PMCPMC3688083.

2. Lee E, Choi EK, Han KD, Lee H, Choe WS, Lee SR, et al. Mortality and causes of death in patients with atrial fibrillation: A nationwide population-based study. PLoS One. 2018;13(12):e0209687. doi: 10.1371/journal.pone.0209687. PubMed PMID: 30586468; PubMed Central PMCID: PMCPMC6306259.

3. Melgaard L, Gorst-Rasmussen A, Lane DA, Rasmussen LH, Larsen TB, Lip GY. Assessment of the CHA2DS2-VASc Score in Predicting Ischemic Stroke, Thromboembolism, and Death in Patients With Heart Failure With and Without Atrial Fibrillation. JAMA. 2015;314(10):1030-8. doi: 10.1001/jama.2015.10725. PubMed PMID: 26318604.

4. Lau WCY, Li X, Wong ICK, Man KKC, Lip GYH, Leung WK, et al. Bleeding-related hospital admissions and 30-day readmissions in patients with non-valvular atrial fibrillation treated with dabigatran versus warfarin. J Thromb Haemost. 2017;15(10):1923-33. doi: 10.1111/jth.13780. PubMed PMID: 28748652.

5. Ando T, Ooba N, Mochizuki M, Koide D, Kimura K, Lee SL, et al. Positive predictive value of ICD-10 codes for acute myocardial infarction in Japan: a validation study at a single center. BMC Health Serv Res. 2018;18(1):895. doi: 10.1186/s12913-018-3727-0. PubMed PMID: 30477501; PubMed Central PMCID: PMCPMC6260564.

6. Schwarzkopf L, Wacker M, Ertl J, Hapfelmeier J, Larisch K, Leidl R. Impact of chronic ischemic heart disease on the health care costs of COPD patients - An analysis of German claims data. Respir Med. 2016;118:112-8. doi: 10.1016/j.rmed.2016.08.001. PubMed PMID: 27578479.

7. Davis JD, Olsen MA, Bommarito K, LaRue SJ, Saeed M, Rich MW, et al. All-Payer Analysis of Heart Failure Hospitalization 30-Day Readmission: Comorbidities Matter. Am J Med. 2017;130(1):93 e9- e28. doi: 10.1016/j.amjmed.2016.07.030. PubMed PMID: 27592085; PubMed Central PMCID: PMCPMC5482409.

8. Keenan PS, Normand SL, Lin Z, Drye EE, Bhat KR, Ross JS, et al. An administrative claims measure suitable for profiling hospital performance on the basis of 30-day all-cause readmission rates among patients with heart failure. Circ Cardiovasc Qual Outcomes. 2008;1(1):29-37. doi: 10.1161/CIRCOUTCOMES.108.802686. PubMed PMID: 20031785.

9. Enomoto LM, Shrestha DP, Rosenthal MB, Hollenbeak CS, Gabbay RA. Risk factors associated with 30-day readmission and length of stay in patients with type 2 diabetes. J Diabetes Complications. 2017;31(1):122-7. doi: 10.1016/j.jdiacomp.2016.10.021. PubMed PMID: 27838101.

10. Chen G, Khan N, Walker R, Quan H. Validating ICD coding algorithms for diabetes mellitus from administrative data. Diabetes Res Clin Pract. 2010;89(2):189-95. doi: 10.1016/j.diabres.2010.03.007. PubMed PMID: 20363043.

11. Takeuchi M, Kawamura T, Sato I, Kawakami K. Population-based incidence of diabetic ketoacidosis in type 2 diabetes: medical claims data analysis in Japan. Pharmacoepidemiol Drug Saf. 2018;27(1):123-6. doi: 10.1002/pds.4271. PubMed PMID: 28752620.

12. Swanson JO, Vogt V, Sundmacher L, Hagen TP, Moger TA. Continuity of care and its effect on readmissions for COPD patients: A comparative study of Norway and Germany. Health Policy. 2018;122(7):737-45. doi: 10.1016/j.healthpol.2018.05.013. PubMed PMID: 29933893.

13. Annavarapu S, Goldfarb S, Gelb M, Moretz C, Renda A, Kaila S. Development and validation of a predictive model to identify patients at risk of severe COPD exacerbations using administrative claims data. Int J Chron Obstruct Pulmon Dis. 2018;13:2121-30. doi: 10.2147/COPD.S155773. PubMed PMID: 30022818; PubMed Central PMCID: PMCPMC6045902.

14. Rottenkolber M, Voogd E, van Dijk L, Primatesta P, Becker C, Schlienger R, et al. Time trends of period prevalence rates of patients with inhaled long-acting beta-2-agonists-containing prescriptions: a European comparative database study. PLoS One. 2015;10(2):e0117628. doi: 10.1371/journal.pone.0117628. PubMed PMID: 25706152; PubMed Central PMCID: PMCPMC4338187.

15. Hadji P, Klein S, Gothe H, Haussler B, Kless T, Schmidt T, et al. The epidemiology of osteoporosis--Bone Evaluation Study (BEST): an analysis of routine health insurance data. Dtsch Arztebl Int. 2013;110(4):52-7. doi: 10.3238/arztebl.2013.0052. PubMed PMID: 23413388; PubMed Central PMCID: PMCPMC3570954.

16. Haussler B, Gothe H, Gol D, Glaeske G, Pientka L, Felsenberg D. Epidemiology, treatment and costs of osteoporosis in Germany--the BoneEVA Study. Osteoporos Int. 2007;18(1):77-84. doi: 10.1007/s00198-006-0206-y. PubMed PMID: 17048064.

17. Adelborg K, Christensen LB, Munch T, Kahlert J, Trolle Lagerros Y, Tell GS, et al. Positive predictive values of International Classification of Diseases, 10th revision codes for dermatologic events and hypersensitivity leading to hospitalization or emergency room visit among women with postmenopausal osteoporosis in the Danish and Swedish national patient registries. Clin Epidemiol. 2017;9:179-84. doi: 10.2147/CLEP.S126370. PubMed PMID: 28392715; PubMed Central PMCID: PMCPMC5373846.

18. Institut für das Entgeltsystem im Krankenhaus GmbH (InEK), Siegburg, Germany. DEUTSCHE KODIERRICHTLINIEN, Allgemeine und Spezielle Kodierrichtlinien für die Verschlüsselung von Krankheiten und Prozeduren. Version 2019. https://www.g-drg.de/Archiv/DRG_Systemjahr_2019_Datenjahr_2017#sm5 (last accessed 02 December 2020).
